# Terahertz beat oscillation of plasmonic electrons interacting with femtosecond light pulses

**DOI:** 10.1038/srep18902

**Published:** 2016-01-06

**Authors:** Xinping Zhang, Jianfang He, Yimeng Wang, Feifei Liu

**Affiliations:** 1 Institute of Information Photonics Technology and College of Applied Sciences, Beijing University of Technology, Beijing 100124, P. R. China

## Abstract

Plasmon resonance in nanostructured metals is in essence collective oscillation of free electrons, which is driven by optical electric fields and oscillates at nearly the same frequency as the excitation photons. This is the basic physics for the currently extensively interested topics in optical metamaterials, optical switching, and logic optical “circuits” with potential applications in optical communication and optical computation. We present here an interference effect between photons and plasmon electrons, which is observed as multi-cycle beat-oscillation. The beat frequency is in the range of 3~4 THz, which is equal to the difference between optical frequency of the photons and oscillation frequency of the plasmon electrons. Such beat oscillation evolves in a time scale of more than 1 ps, which is much longer than the optical pulse length, implying interaction between photons and pure damping plasmon-electrons. The discovered mechanisms might be important for exploring new approaches for THz generation.

Beat frequency oscillation has been commonly observed with acoustic^1^,^2^ and optical fields^3^,^4^,^5^, where the beating is in fact interference between two fields of slightly different frequencies (ω_1_ and ω_2_) and oscillates at a different frequency of ω_3_ = |ω_1_ − ω_2_|. A typical heterodyne-beat effect involving optical electric fields is the frequency-comb technique^6^,^7^,^8^,^9^, where the comb may be widened at least to an octave, the second harmonic generation (SHG) at the lower-frequency side may have almost the same wavelength as the higher-frequency side, so that a heterodyne beat can be generated due to the interaction between them^10^. Similar effect can also be detected due to difference frequency generation (DFG)^11^,^12^,^13^, however, this time the heterodyne beat is generated at the lower-frequency side. Thus, optical beat frequency generation is important in frequency comb techniques and have applications in optical metrology, optical atomic clocks, and high-precision spectroscopy, etc.

Optical excitation of metallic nanostructures may induce collective oscillation of free electrons in a specific spectral range, which is defined as plasmon resonance^14^,^15^,^16^. However, electron-phonon and phonon-phonon coupling processes are inevitably excited simultaneously with the plasmonic electron-electron interactions due to direct thermal excitation and energy transfer from electrons to the lattices^17^,^18^. These processes can be recognized and resolved by ultrafast spectroscopy, since they have different time scales in evolution dynamics and different dependence on the excitation intensity. The electron-electron interactions generally evolve in a time scale shorter than 100 fs^18^,^19^. The electron-phonon coupling decays in a time scale from hundreds of femtoseconds to picoseconds, whereas, the phonon-phonon coupling decays in tens or even hundreds of picoseconds^18^,^19^,^20^. The electron-phonon coupling process slows down the decay dynamics of the plasmonic electrons, so that further electron-photon interaction processes may be resolved into details.

We present here observation of interference interaction between photons and plasmonic electrons, which leads to beat-oscillation in the transmission of femtosecond probe photon though the “hot” plasmonic electron gas. The plasmonic electron-phonon coupling extended decay dynamics to evolve longer than the beat-oscillation cycle, allowing us to observe multiple cycles of the time-domain interference effect with tuning the delay between the femtosecond pump and probe pulses.

Beat frequency oscillation observed as interference between two waves with slightly different frequencies results from alternative “in-phase” and “out-of-phase” interactions between these two waves. Such “phase-matching” mechanisms also apply to the interaction between a femtosecond light pulse and the oscillating electrons in the plasmonic nanostructures driven by another femtosecond pulse. Fig. 1(a) demonstrates collective oscillation of the free-electron gas in a gold nanoparticle when being excited by a few-cycle light pulse (red curve). Figure. 1(b,c) show a probe pulse for in-phase (blue curve) and out-of-phase (green curve) interaction with the plasmonic electrons in (a). “In-phase” interaction implies the plasmonic electrons and the probe photons oscillates at nearly the same phase at the center frequency within the ultrashort pulse duration, whereas, in “out-of-phase” interaction, there is a π phase-shift between the two oscillation processes. For in-phase interaction, the probe photon oscillating in femtosecond scale is more strongly absorbed and scattered by the resonantly excited electron gas. In contrast, the out-of-phase interaction induces reduced absorption and scattering of the probe photons. The above “enhancement” and “reduction” effects for the “in-phase” and “out-of-phase” interactions, respectively, are evaluations in comparison with the case without any fixed phase-relationship between the light field and oscillation of the plasmonic electrons.

Complicated evolution dynamics of plasmonic electrons needs to be resolved for understanding our proposed mechanisms and experimental observations in the beating between the photons and plasmonic electrons. In essence, strong optical excitation changes the distribution of the conduction-band electrons close to the Fermi level, which may result both from the hot conduction-band electrons and from the interband excitation of electrons from the *d* band to the conduction band. The change of the electron distribution gives rise to the modification on the metal’s complex dielectric constant ε_M_(ω, t) both in the real and in the imaginary parts. The modulation in imaginary part Im(ε_M_) is the most significant contribution to the transient response of localized surface plasmon resonance^21^,^22^.

Bigot *et al*.^19^ presented comprehensive analysis on the electron dynamics in metallic nanoparticles both experimentally and theoretically, where the electron-electron scattering and energy relaxation to the lattice were taken as the two main mechanisms to determine the transient electron dynamics. The modeling is based on the energy transfer from the plasmonic hot electrons to the lattices via electron-phonon interactions adiabatically. The distribution of occupied electronic energy states were described by Fermi-Dirac statistics at a specific hot-electron temperature *T*_*e*_. The transient optical response of the plasmonic electrons is considered to be related dominantly to temperature difference between the hot electrons and the lattices, *T*_*e*_(*t*) − *T*_*l*_(*t*), where *T*_*e*_(*t*) and *T*_*l*_(*t*) are the transient electron- and lattice-temperatures under the excitation by a ultrashort pump pulse^23^. Such an approach has been defined as the two-temperature model^19^.

The two-temperature model was first introduced by Kaganov *et al*.^24^ and has been employed extensively in the modeling on the photophysical processes involving electron dynamics in metallic nanostructures, which is based on the energy transfer from the hot electrons to phonons through inelastic collision and thermal diffusion^21^,^22^,^23^,^24^,^25^,^26^,^27^. This model reads:


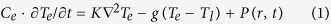



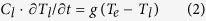


where *C*_*e*_ and *C*_*l*_ are the specific heat capacities of the electron and the lattice, respectively, *K* is the thermal conductivity, *g* is the electron-phonon coupling constant as defined in ref. 23, *P*(*r*, *t*) represents the light-pulse excitation for the heating the electrons. In (1), the temperature dependence of *C*_*e*_ was neglected considering small thermal perturbation^23^. Further reasonable approximation by neglecting the slow thermal conductivity term and the heating source term when considering a femtosecond excitation pulse leads to a simplified expression for the coupled nonlinear differential equations:^23^


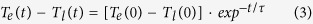


where *T*_*e*_(0) and *T*_*l*_(0) are the initial electron and lattice temperatures following the excitation by a femtosecond light pulse, τ = (*g*/*C*_*e*_ + *g*/*C*_*l*_)^−1^ is the lifetime of the hot electrons in their interaction with the phonons.

In our case, we employed a femtosecond pulse at about 800 nm, which is much smaller in photon energy than that required for the excitation of interband transitions. Therefore the modeling in equations (1)–(3), 2, 3 applies well to our system that involves the collective interactions between conduction-band electrons and inelastic collisions between the electrons and phonons. Moreover, the lifetime of the hot electrons, which was defined using the electron-phonon coupling constant and the electron/phonon heat capacities, actually evaluates the time scale of interactions involved in the electron-phonon system. Therefore, it is reasonable to term it as the lifetime of the electron-phonon interactions (τ_*e−ph*_).

Furthermore, for our gold nanoparticles smaller than 100 nm excited using a femtosecond pulse at about 800 nm, it is reasonable to approximate the electro-magnetic response of them as a dipole oscillating at the same frequency (wavelength) as the excitation optical electric field^28^,^29^. Additionally, the collective electron oscillation dynamics can be well described by a driven and damped harmonic oscillator^28^. As a result, the oscillation of the center-of-mass of the electron cloud has the same frequency as the driving optical electric field, although an amplitude damping and a phase shift may be introduced. In particular, we should consider that the pump wavelength of about 800 nm is within the plasmon resonance spectrum of the gold nanoparticles, it is also reasonable to assume the plasmonic electrons to oscillate at the same frequency as the driving pulses with a constant phase-shift.

Thus, excited by a pump pulse at a wavelength of λ_*1*_ and a pulse length of τ_*P*_, the plasmonic electrons have a temperature with respect to the lattices [*T*_*e*_(*t*) − *T*_*l*_(*t*)] that is proportional to:


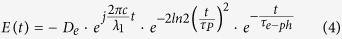


In equation (4), we used a Gaussian function to describe the shape of the pump pulse and have taken into account the fact that the electron oscillation has a π-shift with respect to the optical electric field, where a minus sign is inserted into the complex amplitude of the optical electric field. *D*_*e*_ is simply a constant to define the amplitude of the oscillation.

In the probing process, we sent a light pulse with an equal pulse length of τ_P_ while centered at a different wavelength of λ_*2*_ to the hot-electron-phonon system, which may be characterized as:


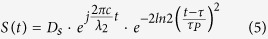


where this probe pulse is delayed by τ from the pump and *D*_*s*_ is a constant to define the amplitude. Thus, due to the modulation by the plasmonic electrons, the transient interaction between the probe pulse and the excited gold nanoparticles is proportional to 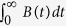 with:


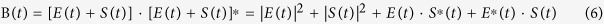


where the stars “*” denote complex conjugates and the integral enables evaluation covering the full pulse length.

In the analysis of the beating effect, we are only interested in the cross terms of 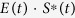 and 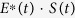, which exert modulation on the direct-current terms and are account for the transient absorption signals. Therefore, we focus our studies on the modulation effects characterized by the cross terms in (6), where we have:


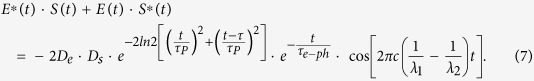


Thus, the transient absorption spectroscopic signals will be proportional to 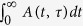 with:


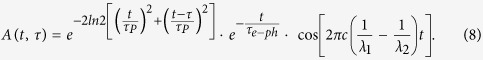


Clearly, the modulation is an oscillation at a circular frequency of *f*_*B*_ = 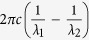 or at a wavelength of 
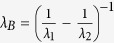
, which is a function of the time delay τ between the excitation and probe light pulses. This simple modeling explains the beat-oscillation of the interaction between femtosecond-pulsed photons and localized surface plasmon resonance (LSPR) in gold nanoparticles, the beat frequency equals the difference between the oscillating frequency of the plasmonic electrons and the optical frequency of the probe photons.

It should be noted that the phase-matching scheme involving in-phase and out-of-phase interactions requires sufficiently close frequencies or wavelengths of the two involved fields and sufficiently short pulse duration. In the following discussions, we fix the pulse length at 150 fs at FWHM for both the pump and probe pulses, which is the same as that we used in the experiments. It is reasonable to assume that the LSPR electrons are oscillating at the same wavelength as the pump during excitation. As demonstrated in simulation results in Fig. 2(a), the pump and probe pulses have a wavelength of 750 and 850 nm, respectively, implying a wavelength difference as large as Δλ = 100 nm. The plot of *B*(t) for in-phase interaction is shown in the right panel of Fig. 2(a). Due to the large value of Δλ, the beat cycle that may be calculated by 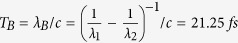 is much smaller than the pulse duration. As a result, the value of 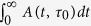 was calculated to be 0, where τ_0_ corresponds to a time delay of peak-to-peak overlap between the excitation and probe light pulses. Apparently, we will also have 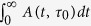 = 0 for out-of-phase interaction. This implies that the “in-phase” or “out-of-phase” processes have no difference when the beat cycle is much smaller than the pulse duration. However, when we reduce Δλ such that the pump and the probe pulses are located at 800 and 810 nm, respectively, with Δλ = 10 nm, *T*_*B*_ is calculated to be 216 fs, which is much larger than the pulse duration of 150 fs. Consequently, the in-phase and out-of-phase processes play completely different roles in modifying the photon-electron interaction, as shown in Fig. 2(b). For the “in-phase” interaction, positive values dominate the plot of *A*(*t*, τ_0_), implying 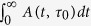 > 0. In contrast, for out-of-phase process, *A*(*t*, τ_0_) shows dominant negative values, resulting in 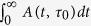 < 0. As has been discussed above, 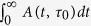 > 0 and 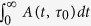 < 0 correspond to the enhanced and reduced optical extinction of probe photons by the oscillating plasmon electrons, respectively. With tuning the delay (τ) between the probe and the driving pump pulses, the value of 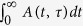 will “oscillate” between the peak positive and negative values, resulting in the beat oscillation of the value of 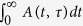. Thus, we should be able to observe periodic variation of the optical extinction of the probe photons by the oscillating plasmonic electrons at a beat wavelength of 
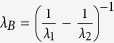
.

To verify this, we performed simulation on the interaction between a 150-fs probe pulse at 810 nm and the plasmonic electron-phonon coupling that is driven by a 150-fs pump pulse at 800 nm. To make the simulation more closely analogous to the practical experiments, we use the electron-phonon coupling process to simulate the evolution dynamics of plasmonic electrons, which is the factual case when the gold particles are excited by intensive femtosecond pulses. We assume a lifetime of 500 fs (τ_*e−ph*_ = 500 fs) for the electron-phonon coupling process. Figure 2(c) shows schematically the interaction between the probe pulse and electron-phonon coupling system at varying time delay of τ. The dashed green curve in Fig. 2(c) is nothing more than a guide to indicate collective oscillation of the free electrons in the gold nanoparticles at the same frequency as the driving pump pulse, since optical frequency at 800 nm is so high that it is difficult to resolve the cycles of the optical electric field of the pump pulse.

Then, 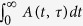 was calculated with tuning the time delay τ and the result is shown in Fig. 2(d). Clearly, the value of 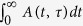 oscillates between positive and negative values, which decays with the damping of the electron-phonon coupling process. Using the calculated curve in Fig. 2(d), we can measure the cycle of the beat oscillation, which is about 280 fs and is larger than the theoretical value of 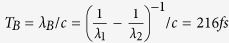. This is because we have included the electron-phonon coupling and thus extended the lifetime of pure plasmonic electrons. The simulation results verified our proposed mechanisms of the beat oscillation in the *LSPR* electron-photon interaction and indicate that the beat frequency equals the difference between the optical frequency of the probe photon and the oscillation frequency of the *LSPR* electrons.

In the experiments, chemically synthesized gold nanoparticles^30^ with a diameter ranging from 5 to 10 nm were dissolved in xylene at a concentration of 100 mg/ml to prepare the colloidal solution, which are covered with ligands to ensure excellent dispensability. The colloidal solution was then spin-coated onto a fused-silica substrate with a thickness of 1 mm and an area of 15 × 15 mm^2^. Then, the sample was annealed in a Muffel furnace at a temperature of about 400 °C for 20 minutes before it was cooled down to room temperature. Thus, a single-layer matrix of randomly distributed gold nanoparticles were achieved. The scanning electron microscopic (SEM) image is shown in Fig. 3(a) with the atomic force microscopic (AFM) image included in the inset, which shows a mean diameter of about 100 nm and a height of more than 70 nm for the gold nanoparticles on the fused silica substrate. The plasmon resonance of the gold nanoparticles is centered at about 670 nm and extends from 500 to longer than 950 nm, as shown in Fig. 3(b).

Transient absorption (TA) spectroscopic measurements were performed using a femtosecond pump-probe system on the gold-nanoparticle matrix, as illustrated schematically in the inset of Fig. 3(b). A Ti:Sapphire amplifier from Coherent Inc. (Legend Elite) provides pump pulses centered at about 798 nm with a bandwidth at FWHM of 5 nm, pulse length of about 150 fs, a pulse energy of 1 mJ, and a repetition rate of 1 kHz. A portion of the 800-nm pulses was focused to heavy water with a thickness of 1 mm to produce supercontinuum pulses extending from 340 to 1200 nm, which were used as the probe in the TA measurements. The pump was focused onto the sample with a diameter of about 0.5 mm and the pump fluence was limited to 650 μJ/cm^2^, ensuring no damage to the gold-nanoparticles. The probe beam was focused into a spot of about 100 μm in diameter within the pump spot on the sample.

The main reasons why we choose an pump position on the falling edge of the optical extinction spectrum include: (1) We should study the beat-oscillation effect with a high contrast at a spectral position relatively far away from that of the most sensitive *LSPR* response, so that the broadening and red-shift of plasmon resonance under optical excitation has little influence on the studied effect. (2) As will be discussed below, the obviously blue-shifted beat-oscillation verifies convincingly not only the interference effect in the photon-electron interactions, but also a tendency of the electrons to oscillate at the frequency of intrinsic plasmon resonance after turn-off of the excitation pulse. (3) This is a most convenient spectral position for a Ti:Sapphire laser system.

The lower panel of Fig. 3(c) shows the TA-measurement data at a pump fluence of 310 μJ/cm^2^, which are plotted by Δ*A* in optical density as a function of wavelength (λ) and delay (τ) between the pump and probe pulses in the spectral range from 720 to 840 nm. If looking along the delay axis, we observe an obvious oscillating behavior of Δ*A* with different cycles at different wavelengths. Furthermore, two spectral ranges are observed on either side of the center wavelength of the pump spectrum at 798 nm, as marked out by a dashed white line. The value of Δ*A* remains nearly zero at 798 nm along the whole delay line. It is understandable that no beat oscillation is excited when the two fields are oscillating at the same frequency or wavelength. The upper panel of Fig. 3(c) presents TA spectra at four delays of 160, 280, 410, and 520 fs, which correspond to the four spectra marked by dashed red lines at ①, ②, ③ and ④ in the 3D plot of the measurement data in the lower panel. The TA spectra exhibit narrower and more dramatic features at wavelengths longer than 798 nm.

According to Fig. 3(c), the beat-oscillation behavior is observed in a much broader spectral range for wavelengths shorter than 798 nm, which extends from 730 to 795 nm. In contrast, the longer-wavelength range for beat-oscillation is observed from 801 to 840 nm. According to Fig. 3(c), the beat-oscillation behavior is observed in a much broader spectral range for wavelengths shorter than 798 nm, which extends from 730 to 795 nm. In contrast, the longer-wavelength range for beat-oscillation is observed from 801 to 840 nm. To understand this, we need to consider the decay dynamics of the plasmonic electrons after turn-off of the excitation pulse at an off-resonance wavelength.

It is understandable that frequency of plasmon resonance is intrinsically determined by the material and structural properties of the nanoparticles. Although the plasmon electrons were forced to oscillate at the excitation frequency within the pump pulse length, they tend to de-phase to the intrinsic oscillation after turn-off of pumping. According to Fig. 2(b), *LSPR* of the gold nanoparticles is centered at about 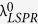 = 670 nm, implying that the gold nanoparticles intrinsically favor collective oscillation of the free electrons at a wavelength of 670 nm, which is much shorter than the center wavelength of the pump pulse at 798 nm. Therefore, after the off-resonance excitation at 798 nm stops, the oscillation of the plasmonic electrons tend to move toward 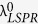. This explains why lowest wavelength observed for beat oscillation may reach 730 nm, which corresponds to interaction with pure de-phasing plasmon electrons. This also confirms interaction between photons and pure damping plasmonic electrons.

Figure 4(a) shows the dynamics of the beat-oscillation process at a series of wavelengths from 736 to 833 nm, which were measured at a pump fluence of 650 μJ/cm^2^. On most of the dynamic curves, we observe more than two-cycles of beat oscillation with each cycle varying from 250 to about 330 fs in time scale, or from 75 to 96 μm in wavelength, or from 4 to 3.125 THz in frequency. This kind of beat oscillation takes about 1 ps before the phonon-phonon coupling process dominates the TA dynamics, which is much longer than the pulse length and lifetime of plasmon resonance due to electron-phonon coupling. The downward triangles in Fig. 4(a) indicate beat-oscillation cycles at 760 nm and at 815 nm. According to 
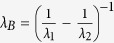
, at a probe wavelength of λ_2_ = 815 nm, the beat-oscillation cycle is measured to be about *T*_*B*_ = 292 fs in Fig. 4(a), corresponding to a frequency of about *f*_B_ = 3.42 THz or a beat wavelength of λ_*B*_ = 87.6 μm, and the wavelength of electron-oscillation can be calculated to be λ_1_≈807.4 nm. However, for λ_2_ = 760 nm, *T*_*B*_ is measured to be 332 fs, then, the corresponding calculation results for the beat oscillation will be: *f*_B_ = 3.01 THz and λ_*B*_ = 99.6 μm, leading to λ_1_≈765.8 nm. Apparently, λ_1_ is within the pump spectrum for λ_2_ = 815 nm and it is outside the pump spectrum for λ_2_ = 760 nm. This implies that probe pulses at λ_2_ = 815 nm interacted with electrons excited within the resonance spectrum, whereas, those at λ_2_ = 760 nm interacted with pure de-phasing electrons after turn-off of the pump pulse.

Figure 4(b,c) demonstrate the pump-fluence dependence of the beat-oscillation dynamics at 760 and 815 nm, respectively, where we increased the pump fluence from 65 to 650 μJ/cm^2^. The amplitude of beat oscillation is enhanced significantly with increasing the pump fluence for both wavelengths. However, the oscillation within the beat cycle from about 600 to 900 fs is much stronger at 815 than at 760 nm. This is also because the probe at 815 nm interacted with plasmon electrons excited more “resonantly” than at 760 nm. The probe pulse at 760 nm interacted with the pure de-phasing electrons that tended to oscillate at the intrinsic plasmon resonance wavelength. As a result, the dynamics at 760 nm decays faster than at 815 nm. This can also be concluded from Fig. 4(a), when we compared the dynamic curves at wavelengths shorter than 780 nm with those at wavelengths longer than 800 nm.

In conclusion, we discovered interference between photons and plasmonic electrons, which induced terahertz beat oscillation in the transmission of femtosecond pulses through metallic nanoparticles. The beat frequency equals the difference between the optical frequency of probe photons and the oscillation frequency of plasmon electrons. These mechanisms were verified not only by the multi-cycle oscillation in the TA dynamics at probe wavelengths within and in the vicinity of the pump spectrum, but also by the significant blue shift of these “beat-oscillation” features. After turn-off of the femtosecond off-resonance excitation, the oscillation frequency of the pure de-phasing plasmonic electrons moves toward the peak frequency of intrinsic plasmon resonance. Thus, the experimental work in our manuscript presents a direct observation of the beating effects in the interaction between photons and plasmonic electrons, which is important not only for understanding electron dynamics in plasmonic nanostructures, but also for exploring ultrafast optical switching devices with improved performance. Furthermore, these discoveries may facilitate plasmonic physics that can be possibly applied in THz-wave generation through plasmon-aided difference-frequency process.

## Additional Information

**How to cite this article**: Zhang, X. *et al*. Terahertz beat oscillation of plasmonic electrons interacting with femtosecond light pulses. *Sci. Rep*. **6**, 18902; doi: 10.1038/srep18902 (2016).

## Figures and Tables

**Figure 1:**
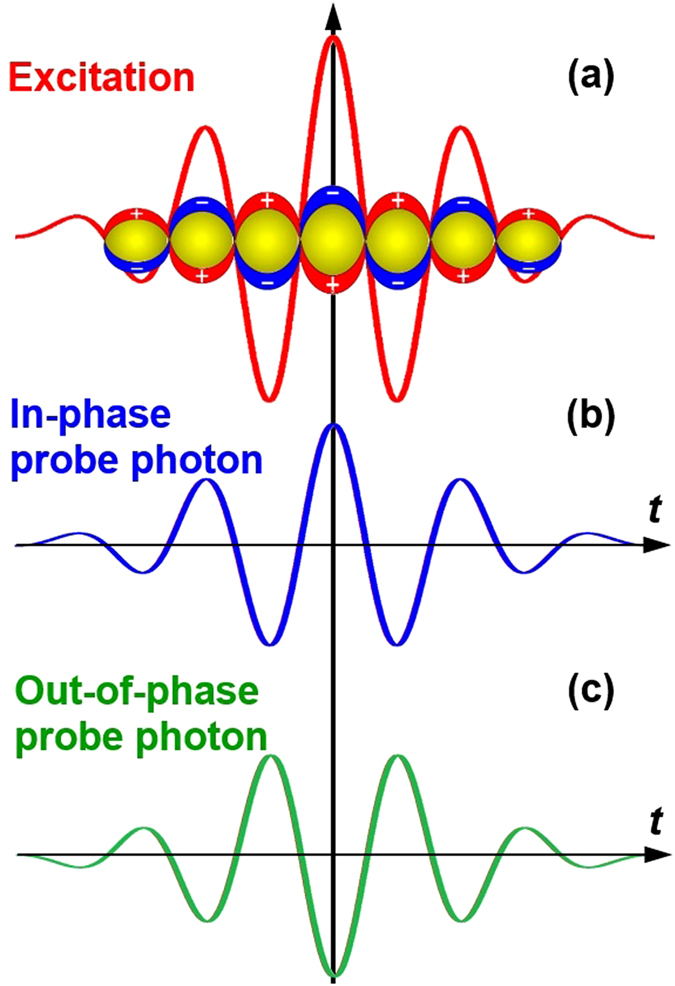
(**a**) Schematic illustration of excitation of transient oscillation of plasmon electrons in a gold nanoparticle using a few-cycle optical pulse. (**b,c**): in-phase and out-of-phase probe photons for coherent interaction with the plasmonic electrons, respectively.

**Figure 2:**
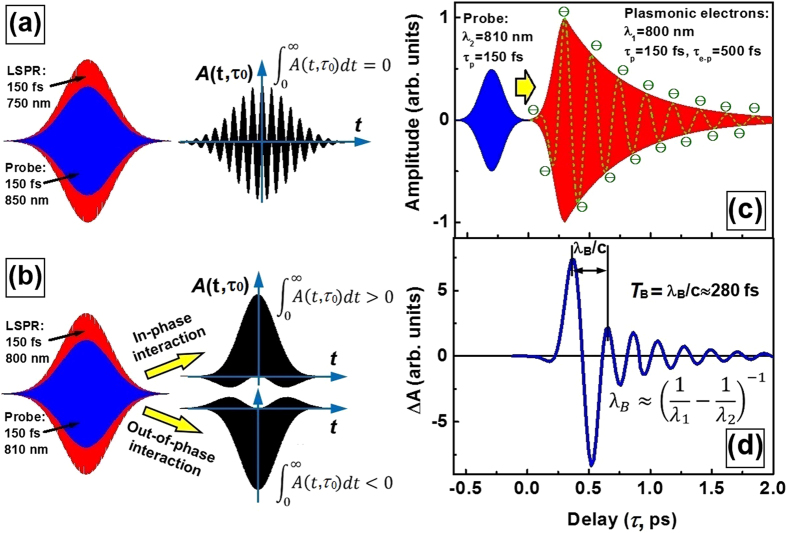
Basic principles of beat oscillation in the interference-interaction between localized surface plasmon electrons and a probe photon. (**a**) Beat-oscillation processes in the interaction within 150-fs between a probe photon at 850 nm and the *LSPR* electrons oscillation at a wavelength of 750 nm. Plot of A(t, τ_0_) indicates a zero value of 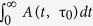, with τ_0_ corresponds to a time delay of peak-to-peak overlap between the excitation and probe light pulses. (**b**) In-phase and out-of-phase interaction between a probe photon and *LSPR* electrons, oscillating at a wavelength of 810 and 800 nm, respectively. The corresponding plots of A(t, τ_0_) indicate a positive value of 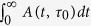 for in-phase interaction and a negative value for out-of-phase interaction. (**c**) Schematic demonstration of the interaction between a 150 fs probe photon pulse at 810 nm and damping oscillation of the plasmonic electron with tuning the delay time, assuming excitation pulse duration of 150 fs with a center wavelength of 800 nm and 1/e lifetime of the electron-phonon coupling of 500 fs. (**d**) Simulation results of the beat-frequency oscillation process, showing a beat cycle of 280 fs that basically agrees with the calculated beat wavelength of 
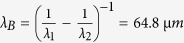
 and beat cycle of 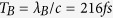.

**Figure 3:**
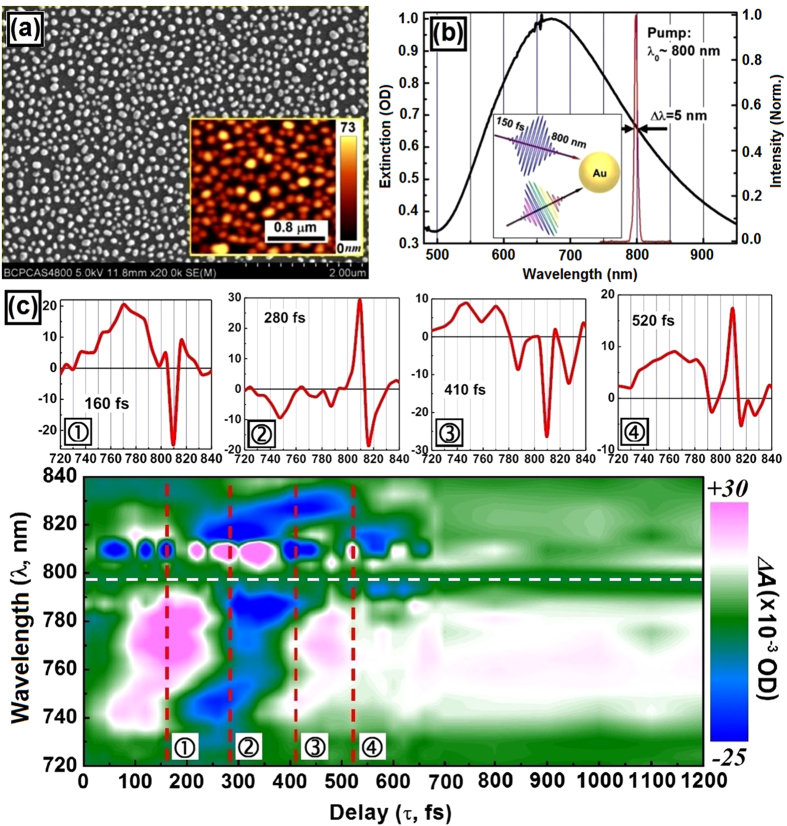
(**a**) SEM image of the random gold-nanoparticles matrix with the AFM image shown in the inset. (**b**) Optical extinction spectrum measured on the gold-nanoparticle matrix and pump pulse spectrum centered at 798 nm. Inset: the pump-probe scheme. (**c**) The transient absorption measurement results presented by Δ*A* as a function of wavelength and delay between the pump and probe pulses. The marks at ①②③④ using dashed red lines in the lower panel correspond to four TA spectra at a delay of 160, 280, 410, and 520 fs shown in the upper panel.

**Figure 4:**
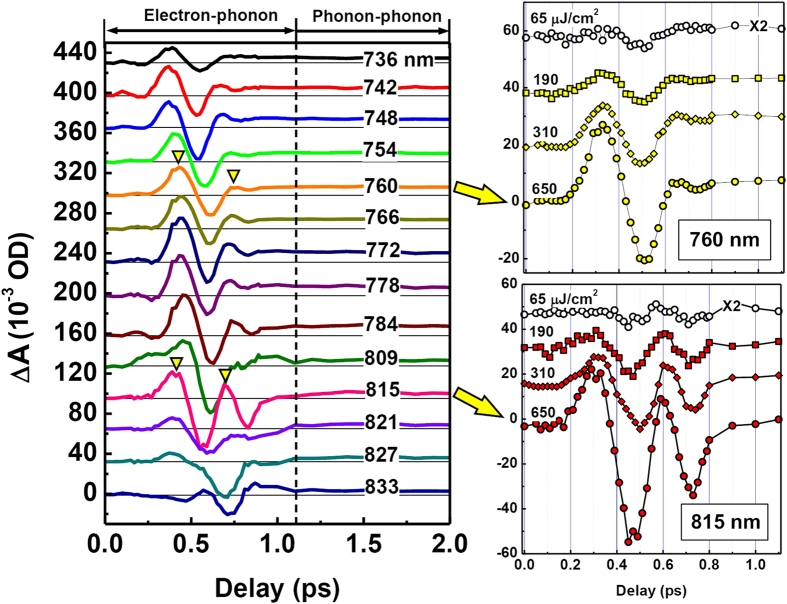
(**a**) TA dynamics at a series of wavelengths within a spectral range from 736 to 833 nm and a time delay range from 0 to 2 ps. (**b,c**): dependence of the TA dynamics at 760 and 815 nm on the pump fluence.
